# The Metropolitan Hospital Saturday Fund

**Published:** 1901-11-09

**Authors:** 


### THE METROPOLITAN HOSPITAL SATURDAY FUND.

The annual public meeting of the Hospital Saturday Fund was held at the Mansion House on Saturday afternoon, 2nd inst., with the Lord Mayor (Mr. Frank Green) in the chair, followed by Mr. R. B. D. Acland, when an urgent message summoned his Worship away. The large hall was full, and the speeches were enthusiastically received. The 27th annual report of the council shows that the total income during 1900 was £20,280, being £267 in excess of the income for the previous year, and £142 more than that of 1897, the last year of the street collections. The fact that, notwithstanding the unprecedented demands on the benevolence of the public, the income of the Fund was more than maintained, indicates, says the report, in an unmistakable manner, the hold which the Fund has on the goodwill of its supporters. The total sum distributed was £17,698. This amount has only been exceeded twice in the history of the Fund, viz., in 1893 and 1896, when the amounts distributed were £17,778 and £18,110 respectively. Thirty-six general hospitals received £6,557; 57 special hospitals received £4,932; and 74 dispensaries, convalescent homes, and miscellaneous institutions received £6,209. The principle of self-help was inculcated with success, as in previous years; in fact, the help afforded by the Fund could not be continued but for the part-pay system of maintaining patients at convalescent homes and of providing surgical appliances. The total number of patients sent to convalescent homes was 1,165. Of this number 812 were sent with free letters, and 353 paid £354 towards the cost of their maintenance. The number of surgical appliances supplied was 3,115, the cost being £2,558; of this sum the recipients paid £1,377, and a grant from the Fund met the remainder. The demand for ambulance boxes for use in workshops was well maintained. There were 75 in use at the end of the year (1900), and, in addition, not a few were purchased. The two divisions of the St. John Ambulance Brigade connected with the Fund, the one for men and the other for women, maintained their efficiency and rendered public service on many occasions. The arrangements with the Invalid Transport Corps of the St. John Ambulance Association continued to work well. The expenses of management amounted to £2,277. Compared with the gross receipts, this sum is at the rate of 11·23 per cent., the lowest rate recorded, with the exception of that for the preceding year, when it was 10·95.

The Lord Mayor gave a sincere and hearty welcome to the audience, all the more hearty, he said, because he fully recognised the benefits of the Fund. It appealed directly to the working men of the Metropolis, who knew, sometimes from sad experience, what its benefits were. Through them the committee was selected, valuable ambulance work was done, and knowledge was disseminated. He was very gratified to find that a further benefit was now to be conferred—namely, a more intimate knowledge of tuberculosis, that most dire disease which, more than any other, increased the annual death-rate. By means of greater knowledge it might be met and eradicated. Those who desired, from their own knowledge and superior scientific skill, to help this forward, were seeking to do so through the dissemination of literature giving information on the subject. In dealing with the Hospital Saturday Fund, he might state the interesting fact that the income of £20,280 for 1900 was almost entirely made up of small weekly contributions. The main principle of the Fund was that it helped those who showed an inclination to help themselves. He ventured to think he would have the entire support of the meeting in saying that that was a very desirable motive. It was this which actuated the committee to propagate information tending to better the condition of the working classes. The figures showed what a very great amount of relief was being given to the suffering poor by an institution that had it in its power to give it. The sum of £1,377 for appliances had been contributed by the patients themselves. The Fund had been in existence for twenty-seven years, and he hoped the record of work would be sufficient to engage the hearty support of all present. The Fund could not possibly continue to prosper unless it was supported by a generous and benevolent public. He hoped he had not said too much; at the same time he hoped he had said enough to gain the earnest support of the public, and that that support would be even greater in the future than it had been in the past.

The Lord Mayor then called upon the Chairman of the Fund, Sir Savile Crossley, to make a statement.

Sir Savile Crossley said this was the first occasion on which, as chairman, he had been able to be present at the annual meeting; last year he was in South Africa. Before entering on the work of the Fund, he might perhaps say a word about a letter sent by the Prince of Wales's Fund to the papers, which stated that an appeal was being sent out to the offices and workshops in London. He wanted to make it clear that this did not trespass on the Hospital Saturday Fund, because the names of those who already subscribed to that Fund had been cut out, and the appeal had been sent to the rest. The fact was that a list of those who subscribed to the Saturday Fund had been sent at the beginning to the Prince of Wales's Fund, and a great many subscribed; but after the first year a large number, finding they could not get letters from the Prince of Wales's Fund, became subscribers to the Saturday Fund. This showed, he thought, that there had been no trespass. There was one other matter upon which he would touch; it was one that he hoped to bring before the executive committee, and that was the date of the annual meeting. It would, he thought, be more satisfactory to have it when the accounts were made up, and these, like those of all business concerns, were wound up early in the year. He only wished to hint at this however, as it was a matter that should be dealt with by the executive. The main object of the Fund was to enable the wage-earners to subscribe to the hospitals, and it represented a far larger number of subscribers than any other hospital fund. It had certain advantages. Some hospitals were able to get more money than others; their facilities depended on where they were situated. Those that were in a good position were apt to get more than others. Then, again, a man might be an excellent secretary, he might keep the hospital books in first-rate order, but he might not be able to bring in large sums in subscriptions. Another advantage was that protests on hospital management had far greater weight coming from a large body of subscribers than if they proceeded from a single individual. For some reason the public were averse to reading printed reports; they were, however, willing, he did not know why, to listen to speeches; and he would like to say one word with regard to the various branches of the Fund. There was, first, a surgical appliance branch. The instruments were made by makers specially employed by the Fund. Except in special cases, the patients were expected to pay part of the cost. Secondly, there was the ambulance work. The Ambulance Committee formed a centre for the St. John Ambulance Brigade. This department provided boxes of First Aid appliances, which were sent to workshops where there was a man who held a certificate for First Aid. Thirdly, there was the nursing department; grants were made according to the number of visits paid by the nurses. He would mention a case in which great expense had been saved by employing nurses: the children of an employee at the Royal Arsenal had attended an ophthalmic hospital, where it was found necessary to put drops periodically into their eyes; the railway fare was 2s. 4d.; but by arranging with the district nurse to attend them, further visits to the hospital were rendered unnecessary. Fourthly, there was the *Quarterly Journal*, about which he would only say that special efforts had been made through its means during the last twelve months to promote a knowledge of tuberculosis. Fifthly, there were the local committees, which were formed in the various districts of London; since the abolition of the street collection, these had done splendid work. Sixthly, the convalescent homes, in which, as in every other department, the principle of self help was inculcated; patients, where possible, paid half the cost. He thought it was quite clear that very great discrimination was used by the committees, and the saving to hospitals was enormous, since a great many patients were found not to be so ill as they thought, and thus it was unnecessary to send them to the hospitals, where the out-patients' departments were always crowded. This weeding out was an important and necessary work.

Mr. Richard H. Green, J.P., Mayor of Poplar, then moved the following resolution:—"That this meeting earnestly appeals to employers and employees to co-operate together and with the Hospital Saturday Fund for the further extension of small, regular, weekly contributions in aid of the medical charities of London, and trusts that special efforts will be made in the future for the further extension of this system." Mr. Green, who was greeted with applause, said he put forward the motion with very great pleasure, for he was entirely in sympathy with it. Personally, he had always been opposed to the street collection, but the advocacy of local committees was a very different thing, and he might say that he stood on that platform as a result of their efforts. Turning to the report, he noticed that out of some 32 local committees, £1,000 was contributed by only eight. Now, if eight could get together £1,000, the remaining 24 should have produced £3,000; he hoped this rule of three sum would be better worked out next year, and that a more equal proportion would appear in the report. The system of local organisation lent itself to friendly rivalry between the various local committees. Since the street collection was given up, the Fund had actually increased, and he would make a special appeal to the working men not to relax their efforts.

Mr. Bousfield seconded the motion, and the resolution was carried unanimously.

The Lord Mayor, being obliged to leave to attend other duties, asked Mr. R. B. D. Acland to fill his place in the chair; but before leaving he called Mr. Charles Austin to come forward. "I have the very great pleasure," said his Worship, when the young man was on the platform, "of handing to you this diploma of merit of the Ambulance Committee, awarded to you for exceptional services in ambulance work. I am very happy that it should have fallen to my lot to present it to you." After presenting the diploma, which was signed by the officers of the Ambulance Committee, the Lord Mayor shook hands with Mr. Austin, and left the platform amid applause.

Mr. Acland said he found himself in the unusual position of having to call upon himself to move a resolution; he should occupy the briefest possible time, as he was sure, if the audience was wise, it was anxious to hear Sir James Crichton Browne on tuberculosis. He had always maintained that the Fund had two distinct duties; first, that of collecting and distributing funds, and secondly that of using its unique position for educational purposes. It had representatives in thousands of workshops, and opportunities of distributing the information placed at its disposal. He asked them not only to pay the very greatest attention to Sir James Crichton Browne, but to help to stamp out that disease which by self-control he believed could be stamped out. The resolution was as follows:—

"That this meeting of representatives of the Hospital Saturday Fund cordially approves the efforts that are being made by the National Society for the Prevention of Tuberculosis, and offers its hearty co-operation in disseminating among the workshops and places of business in London, any information as to means that may be adopted to prevent the spread of this terrible disease."

The motion was seconded by Mr. G. W. Smythe, one of the delegates from the Fund to the International Congress on Tuberculosis.

Sir James Crichton Browne, who was greeted with enthusiasm, supported the resolution, and speaking on behalf of the National Society for the Prevention of Tuberculosis, advocated the co-operation of the Hospital Saturday Fund in its work.

Mr. Acland warmly supported Sir James Crichton Browne's proposals, and moved a vote of thanks to him for his address, after which the meeting terminated.

